# A Biological Comparison of Three *Colletotrichum* Species Associated with Alfalfa Anthracnose in Northern China

**DOI:** 10.3390/plants13131780

**Published:** 2024-06-27

**Authors:** Wennan Zhou, Yanru Lan, Cory Matthew, Zhibiao Nan

**Affiliations:** 1 State Key Laboratory of Herbage Improvement and Grassland Agro-Ecosystems, Lanzhou University, Lanzhou 730020, China; zhouwn19@lzu.edu.cn (W.Z.); lanyr19@lzu.edu.cn (Y.L.); corym@lzu.edu.cn (C.M.); 2 Key Laboratory of Grassland Livestock Industry Innovation, Ministry of Agriculture and Rural Affairs, Lanzhou University, Lanzhou 730020, China; 3 Engineering Research Center of Grassland Industry, Ministry of Education, Gansu Tech Innovation Centre of Western China Grassland Industry, Lanzhou University, Lanzhou 730020, China; 4 College of Pastoral Agriculture Science and Technology, Lanzhou University, Lanzhou 730020, China

**Keywords:** lucerne, *Colletotrichum*, multi-locus, phylogenetic, pathogenicity

## Abstract

Anthracnose caused by various species of *Colletotrichum* is one of the most prevalent diseases in alfalfa worldwide that not only reduces forage yields but also severely compromises forage quality. A comprehensive survey was conducted in 2020 in the main production regions of northern China. The survey results showed that alfalfa anthracnose is prevalent in northern China, with the disease incidence ranging from 9% to 45% and the disease index from 5 to 17 (maximum possible score: 100). In total, 24 isolates were collected and identified as three *Colletotrichum* species (*C. trifolii*, *C. truncatum* and *C. americae-borealis*) based on morphological characteristics and phylogenetic analysis (combined sequences *ITS*, *HIS3*, *ACT* and *GAPDH*). The three species displayed remarkable environmental adaptability, exhibiting a capacity for growth, sporulation and conidial germination in temperatures ranging from 4 to 35 °C and in different nutrient conditions. Pathogenicity assays showed that *C. trifolii* was more virulent than the other two species, although the growth vigor (in terms of colony diameter, sporulation and conidial germination) of *C. truncatum* was the greatest.

## 1. Introduction

Alfalfa (*Medicago sativa* L.) is a globally cultivated perennial legume forage with exceptional agricultural traits and substantial ecological importance. It plays a pivotal role in providing highly nutritive forage for livestock feed, maintaining agricultural sustainability through crop rotation with wheat or maize, and improving soil quality via biological nitrogen fixation [1,2,3]. Disease pressure, particularly from fungal disease, dramatically constrains alfalfa production in many regions. 

*Colletotrichum* is regarded as one of the 10 most important plant pathogenic fungal genera in the world, due to its robust pathogenicity and extensive distribution [4,5]. The presence of anthracnose associated with *Colletotrichum* infection not only has a detrimental effect on crop yields but also severely compromises their quality. In susceptible alfalfa varieties, severe infection can lead to losses of 25–30% in forage yield under conditions favorable to disease spread [6,7]. The forage nutritive quality of infected alfalfa is also significantly affected, with decreased crude protein content (35% reduction) and in vitro digestibility [8,9]. Anthracnose can manifest at any time throughout the entire growing season and on stands of any age [10]; specifically, mortality rates of alfalfa seedlings can even reach 100% in the greenhouse [11]. Typically, the pathogen affects leaves, stems and the crown, but predominantly the stem. Sometimes, under favorable conditions, multiple lesions may appear randomly distributed along the stem, and subsequently, these lesions can enlarge, coalesce and even girdle the stems, ultimately leading to stem death. In such cases, conidia are produced on stems and spread from plant to plant by wind, rain or irrigation [7]. In addition, the anthracnose fungus has the potential to progress downwards from the infected stem into the crown tissues, resulting in reduction in yield of field stands of alfalfa [10]. This infection mode, which leads to *Colletotrichum* crown rot in alfalfa, poses a significant challenge for disease control, particularly in field conditions. Therefore, the etiology and distribution of alfalfa anthracnose require further investigation to comprehensively evaluate the occurrence of this disease in the field. 

Since the initial identification of *Colletotrichum trifolii* as the causative agent of alfalfa anthracnose by Bain and Essary in 1906 [12], research on this disease has been continually ongoing. More recently, several other *Colletotrichum* species have also been associated with this disease, including *C. destructivum* [13], *C. coccodes* and *C. dematium* [14,15], *C. gloeosporioides* [16], *C. truncatum* [17], *C. tofieldiae* [18], *C. lini* [19], *C. spinaciae* [20], *C. americae-borealis* [21] and *C. sojae* [22]. Previously, the *Colletotrichum* species were identified mainly based on their morphological and cultural characteristics. It has been demonstrated, however, that morphological characteristics can vary with culture medium and temperature and are therefore unreliable for species identification in *Colletotrichum* isolates [23]. Taxonomic uncertainties have also posed challenges in diagnosing disease accurately, understanding the host–pathogen relationship, developing resistant varieties and establishing cost-effective field management systems and quarantine programs. The development and utilization of molecular techniques, specifically the comparative analysis of multiple nucleotide sequences, such as the nuclear rDNA internal transcribed spacer (*ITS*) region, actin (*ACT*), histone (*HIS3*) and glyceraldehyde 3-phosphate dehydrogenase (*GAPDH*), have yielded substantial insights into both the phylogenetic relationships among individual populations and the genetic structure of the populations [24]. Previous studies have indicated the efficacy of a phylogenetic approach in conjunction with morphological analyses for discerning the points of difference among closely related *Colletotrichum* species and even among individuals of the same species [21,22,25,26]. Even so, there remains a lack of research on the etiology and ecology of alfalfa anthracnose in major production areas in China, and understanding of the variability and aggressiveness of *Colletotrichum* species isolates from alfalfa remains limited. Therefore, there is a need for a more comprehensive understanding of the fungal species causing alfalfa anthracnose and the biological characteristics of *Colletotrichum* species.

Thus, the objectives of this study were (i) to investigate the distribution and prevalence of alfalfa anthracnose in northern China, (ii) to compare phylogenetic relationships and biological diversity among collected isolates based on multigene sequence data and morphological features and (iii) to compare the adaptability of *Colletotrichum* species to a range of culture conditions and virulence to alfalfa in those differing conditions.

## 2. Results

### 2.1. Distribution, Incidence and Symptoms of Colletotrichum Species

**Figure 1:**
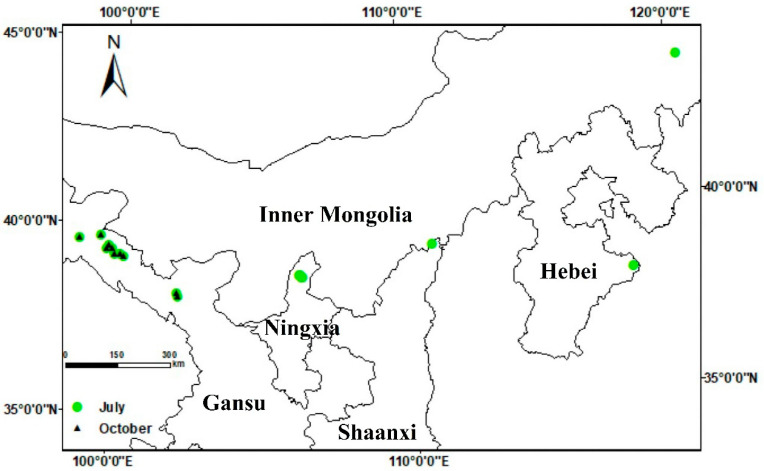
Geographical location of sampling sites within their respective provinces.

Details of the survey sites and alfalfa cultivation and management are summarized in Table 1 and Figure 1. Daily maximum temperatures during July ranged from 26 to 32 °C and daily minima from 13 to 23 °C. In October, maximum daily temperatures ranged from 15 to 19 °C, and daily minima ranged from 5 to 7 °C (Table S1). The weather conditions were favorable for the development of alfalfa anthracnose. Alfalfa anthracnose was prevalent in the primary alfalfa-producing regions in northern China during the survey period, with the exception of the Fugu region in Shaanxi province and the Huanghua region in Hebei province (Table 2). Interestingly, there was no significant occurrence of alfalfa anthracnose detected in surveys in the Helan district of Ningxia province and from Arhorchin in Inner Mongolia. At the latter sites, only a few samples were collected, from which some *Colletotrichum* isolates were obtained. Alfalfa anthracnose was detected in five counties in Gansu province, with the incidence ranging from 9% to 45% and the disease index ranging from 5 to 17 (maximum possible score 100). Among the surveyed sites, those at Suzhou (Gansu province) had the highest disease incidence and index scores (45% and 17, respectively).

**Figure 2:**
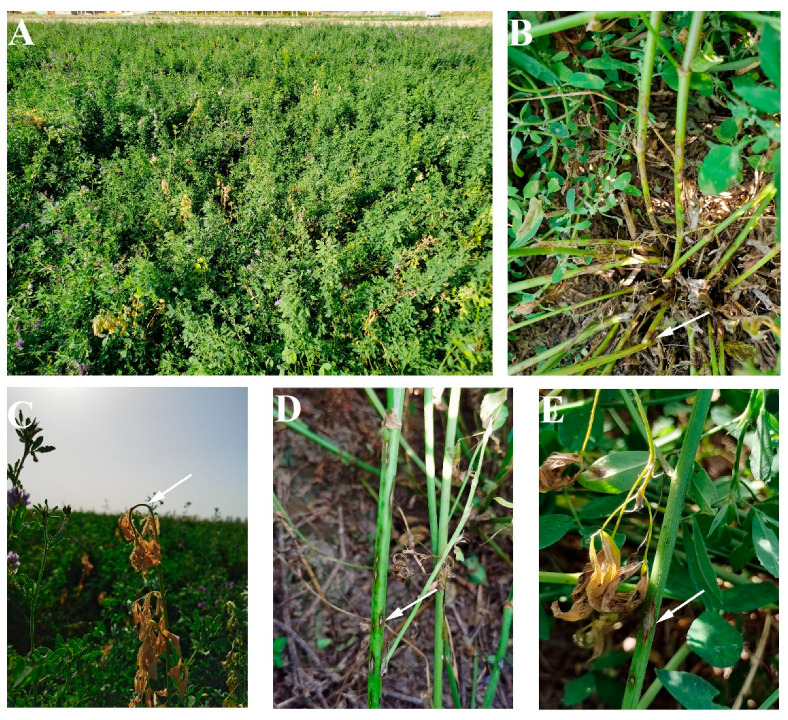
Symptoms of alfalfa anthracnose caused by *Colletotrichum* species under natural conditions: (**A**), an anthracnose-infected alfalfa field; (**B**), the base of the stem easily broken after infection of anthracnose (arrowed); (**C**), Shepherd’s crook shape deformation (arrowed) of the diseased shoot tip; (**D,E**), typical anthracnose lesion (arrowed).

The symptoms of alfalfa anthracnose were easily distinguished from other diseases in the field by the presence of a characteristic ‘shepherd’s crook’ on the stem tip and diamond-shaped spots on the stem (Figure 2A,C). For individual branches, the anthracnose spots commonly occurred on the lower part of the alfalfa stem, and in the early stages of disease development, small black watery spots appeared on the stem; generally, these spots expanded in a diamond-shaped or oval-shaped pattern or irregularly with a black center in the lesion. The lesion progressed to the next stage with an unaltered shape but increased size; meanwhile, the center of the lesion exhibited a grayish-white color, while its border displayed shades of dark brown to black. Additionally, the boundary between diseased and healthy tissue was clearly demarcated (Figure 2D,E). Subsequently, the spots continued to expand, and some lesions merged to become stem-girdling, the leaves of diseased plants turned yellow and showed wilting symptoms, and the diseased stems were easily broken at the location of the lesion (Figure 2B). In the advanced stages of disease development, the entire branch became desiccated but remained erect. Under wet conditions, acervuli of the *Colletotrichum* species were observed at the centers of lesions, producing large numbers of conidia. Spots caused by different *Colletotrichum* species exhibited similar characteristics in the field, and differentiation of these species based solely on characteristics observed in natural field conditions was not possible.

### 2.2. Pathogen Isolation and Morphological Observation

A total of 24 *Colletotrichum* isolates were collected in this research and grouped into three *Colletotrichum* species based on a high similarity of morphological characteristics as well as *ITS*, *ACT*, *HIS3* and *GAPDH* sequences. *C. trifolii* was the predominant species with 14 isolates, followed by *C. americae-borealis* with 6 isolates and *C. truncatum* with 4 isolates. *C. truncatum* was exclusively isolated from Arhorchin (Inner Mongolia), whereas *C. americae-borealis* was found in Helan (Ningxia province), Linze and Suzhou (Gansu province), and *C. trifolii* was identified in Yongchang, Gaotai, Yumen, Linze and Suzhou (Gansu province). It was noteworthy that two species were simultaneously isolated from Linze and Suzhou, implying the possibility of mixed infection in the field. 

**Figure 3:**
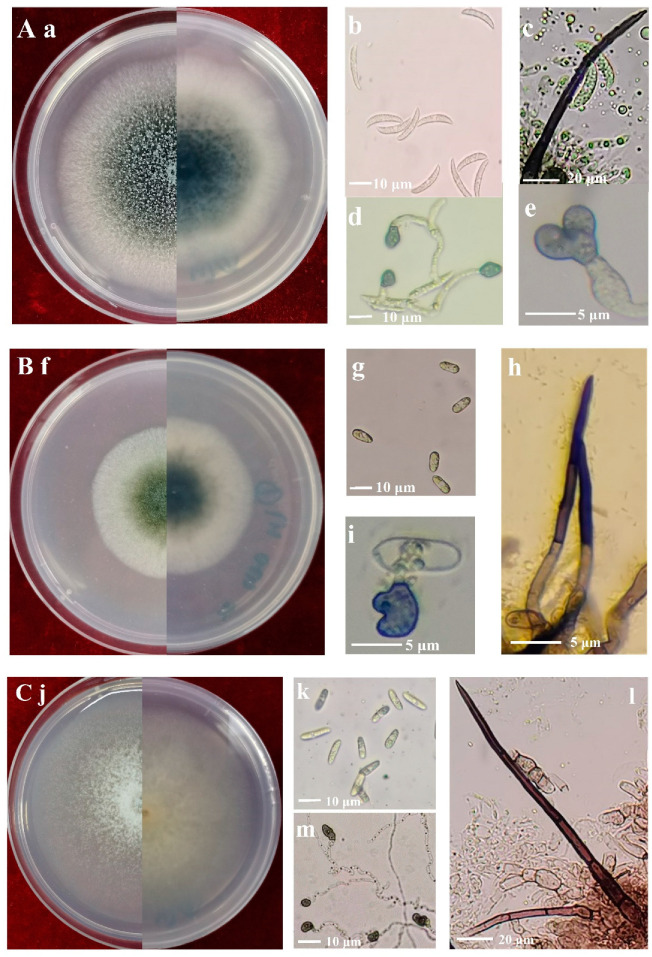
Morphology of *Colletotrichum* species isolated from alfalfa. (**A**), *C. truncatum* (Isolate NMNT22); (**B**), *C. trifolii* (Isolate GTSD07); (**C**), *C. americae-borealis* (Isolate LZBD07); (**a**,**f**,**j**), front and back views of 7-day-old cultures on PDA media; (**b**,**g**,**k**), conidia; (**c**,**h**,**l**), setae; (**d**,**e**,**i**,**m**), appressoria.

The 7-day-old colonies of isolates identified as *C. truncatum* on PDA displayed a dark-green coloration with delicate white margins, while the surface of the colonies appeared rugged and granular (Figure 3a). The conidia were hyaline, smooth-walled to slightly verruculose, aseptate, falcate, one end acute apex, and the other rounded with granular content, 14.0–23.7 × 3.2–7.5 μm, av. ± SD = 19.4 ± 2.1 × 4.8 ± 0.9 μm (Figure 3b). Setae were cylindrical at the base and the tip acute; they varied in color from pale to medium brown, and had a smooth or verrucose wall, 2–5 septate, 32–141 μm long, 1.1–5.5 μm diam (Figure 3c). Appressoria were medium brown, aseptate, solitary, smooth-walled with an elliptical to irregular outline, 2.2–6.8 × 1.7–5.3 μm, av. ± SD = 4.0 ± 1.1 × 3.1 ± 0.8 μm (Figure 3d,e). 

The colonies of isolates identified as *C. trifolii* on PDA after seven days were olivaceous with gray peripheries (Figure 3f). Conidia were cylindrical, aseptate, smooth-walled and hyaline, both ends rounded, 8.1–20.3 × 3.2–9.2 μm, av. ± SD = 12.3 ± 1.5 × 5.4 ± 0.9 μm (Figure 3g). Setae were dark brown to black, smooth-walled, with 1–4 septate, and base cylindrical and tip rounded, 23–57 μm long, and 1.8–5.3 μm diam (Figure 3h). Appressoria were brown, aseptate, solitary, smooth-walled with an elliptical to irregular outline, 5.3–14.8 × 3.8–12.2 μm, av. ± SD = 8.8 ± 1.0 × 6.0 ±1.8 μm (Figure 3i).

The colonies of isolates identified as *C. americae-borealis* on PDA after seven days were faint pink with a diffuse edge (Figure 3j). Conidia were cylindrical or fusiform, aseptate, hyaline, and smooth-walled, both ends rounded, 8.8–18.3 × 3.7–6.9 μm, av. ± SD = 13.5 ± 1.8 × 4.5 ± 0.7 μm (Figure 3k). Setae were dark brown to black, smooth-walled, with 1–4 septate, and base cylindrical and tip rounded or acute, 54–203.4 μm long, 2.3–5.8 μm diam (Figure 3l). Appressoria were brown to dark brown, aseptate, solitary, smooth-walled with an irregular outline, 3.2–10.6 × 2.4–7.8 μm, av. ± SD = 8.0 ± 1.9 × 5.1 ± 0.9 μm (Figure 3m).

### 2.3. Sequence and Phylogenetic Analyses 

**Figure 4:**
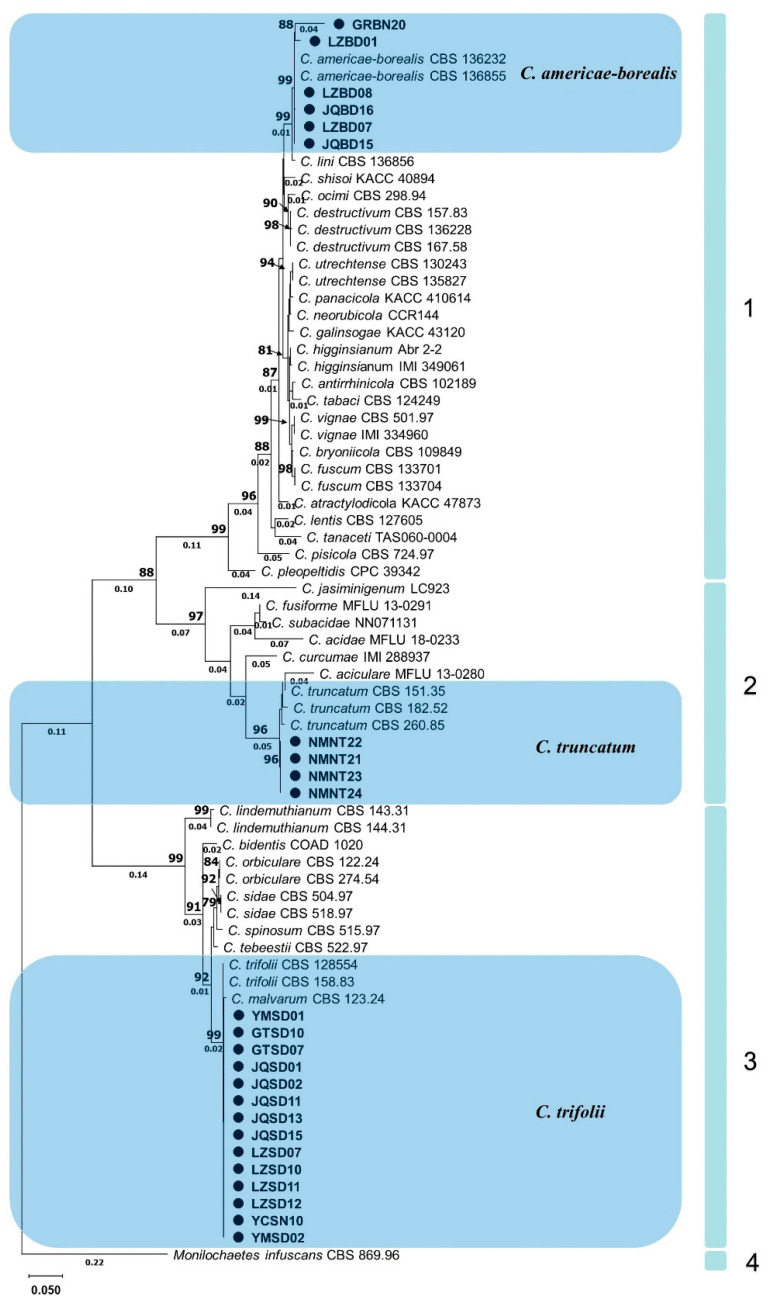
Phylogram generated from maximum likelihood analysis based on sequence alignment of *ITS*, *HIS3*, *ACT* and *GAPDH* of selected *Colletotrichum* species. Isolates obtained in this study are marked with circles. Bootstrap support values above 75% and branch lengths above 0.01 are shown at the nodes.

The individual dataset of *ITS*, *HIS3*, *ACT* and *GAPDH* exhibited consistent tree topology for the 75% reciprocal bootstrap trees, allowing their integration without any conflicts. In the multi-locus analyses of 24 isolates and related *Colletotrichum* species and the outgroup (*M. infuscans* CBS 869.96), positions with less than 50% site coverage were eliminated, resulting in a final dataset containing a total of 1574 positions. The phylogenetic analyses were conducted using both neighbor-joining and maximum likelihood methods, but only the tree generated by the maximum likelihood method is presented (Figure 4).

According to the established multigene phylogenetic tree, 4 main clades and 36 subclades with different *Colletotrichum* species were accepted (Figure 4). The first main clade, *C. destructivum* species complex (bootstrap support value = 99%), consisted of several closely related species including *C. americae-borealis*. A subclade with 99% bootstrap support, formed by isolates GRBN20, LZBD01, LZBD08, JQBD16, LZBD07, JQBD15 and two representatives *C. americae-borealis* isolates (CBS 136232, and CBS 136855), also belonged to this main clade. The second main clade was a *C. truncatum* species complex (bootstrap support value = 97%). A subclade, formed by isolates NMNT22, NMNT21, NMNT23, NMNT24 and three *C. truncatum* isolates (CBS 151.35, CBS 182.52 and CBS 260.85) with 96% bootstrap support, also belonged to the second main clade. The third main clade was the *C. orbiculare* species complex with 99% bootstrap support. A subclade, formed by isolates YMSD01, GTSD10 and 12 other isolates as well as two *C. trifolii* (CBS 158.83 and CBS 128554) with 99% bootstrap support, belonged to this main clade. Clade four contained only one subclade, representing *M. infuscans* (CBS 869.96). 

### 2.4. Biological Characterization at Different Temperatures

**Figure 5:**
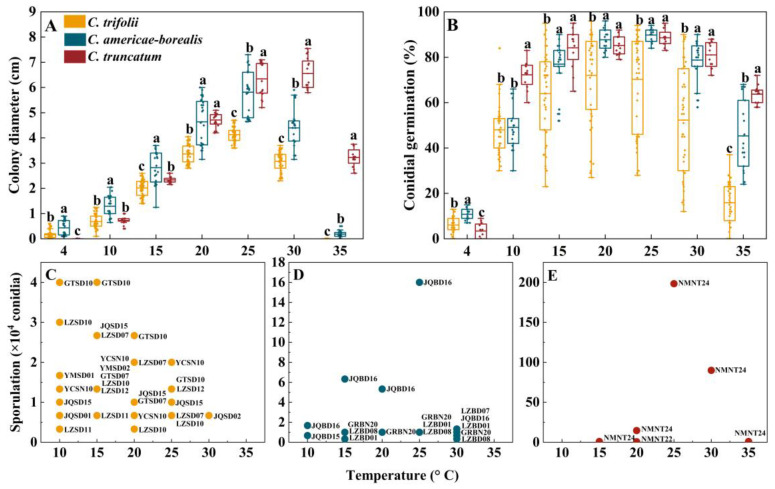
Colony diameter, conidial germination and sporulation for *Colletotrichum* species at different temperatures. (**A**), Colony diameter and (**B**), Conidial germination for the three *Colletotrichum* species (as identified by color in the legend) at different temperatures. Dots for each *Colletotrichum* species are individual isolates. Means with the same letter (a, b, or c) for a given temperature do not differ significantly among the three *Colletotrichum* species by Tukey’s LSD test (*p* ≥ 0.05); (**C**–**E**), Sporulation, for the three *Colletotrichum* species at different test temperatures. The labels are codes of the isolates, and the data are shown in Table S6.

Although the majority of the 24 isolates of the three species were able to grow within a temperature range of 4–35 °C, none of them exhibited growth at 40 °C (Figure 5A). Unexpectedly, *C. trifolii* did not grow at 35 °C and *C. truncatum* did not grow at 4 °C in this study. In the temperature range of 4 to 15 °C, the colony diameter of *C. americae-borealis* was significantly larger than that of *C. trifolii* and *C. truncatum*. When the temperature range was 25 to 35 °C, the colony diameter of the three species ranked as follows: *C. truncatum* > *C. americae-borealis* > *C. trifolii*. These results suggest that *C. truncatum* may be better adapted to slightly higher temperatures than *C. americae-borealis* and *C. trifolii* (Figure 5A). The maximum colony diameter was observed to be 4.1 cm for *C. trifolii* and 5.8 cm for *C. americae-borealis* at 25 °C, while that for *C. truncatum* was 6.6 cm, observed at 30 °C (Figure 5A). 

Conidia germinated at temperatures ranging from 4 to 35 °C; none of them germinated at 40 °C (Figure 5B). The average conidial germination was observed to be 11%, 6% and 3%, respectively, for *C. americae-borealis*, *C. trifolii* and *C. truncatum* at 4 °C, and the differences among the three species were significant (*p* < 0.05). At 10 °C, *C. truncatum* had higher germination than the other two species. For temperatures between 15 and 30 °C, conidial germination ranked *C. truncatum* = *C. ameicae-borealis* > *C. trifolii,* while at 35 °C conidial germination ranked *C. truncatum* > *C. ameicae-borealis* > *C. trifolii* (Figure 5B). The maximum conidial germination was 72% for *C. trifolii* at 20 °C and 90% and 89% at 25 °C for *C. americae-borealis* and *C. truncatum*, respectively.

The temperature range of sporulation of *C. trifolii* and *C. americae-borealis* was 10–30 °C, while that of *C. truncatum* was 15–35 °C (Figure 5C–E). The sporulation of *C. trifolii* was greater than *C. americae-borealis* at 10 °C, and *C. americae-borealis* was greater than *C. trifolii*, followed by *C. truncatum* at 15 °C in this study. At temperatures ranging from 20 to 35 °C, *C. truncatum* had the greatest sporulation, followed by *C. americae-borealis,* and *C. trifolii* had the least sporulation. The maximum sporulation of *C. trifolii* was 4.3 × 10^4^ conidia at 10 °C and 15 °C (Figure 5C), while the maximum sporulation of *C. americae-borealis* was 1.6 × 10^5^ conidia at 25 °C (Figure 5D), and for *C. truncatum*, the maximum sporulation was 2.0 × 10^6^ conidia at 25 °C (Figure 5E). An intriguing phenomenon had captured our attention: not all isolates produced spores in response to the same culture conditions, as evidenced by only seven isolates of *C. trifolii* producing spores at 10 °C. This was only half of the total number of *C. trifolii* in this research (Figure 5C); *C. americae-borealis* and *C. truncatum* also exhibited this phenomenon (Figure 5D,E). 

### 2.5. Biological Characterization in Different Nutrient Conditions

In the ANOVA, the effects of the species, isolate and medium were significant for colony diameter, sporulation and conidial germination (*p* < 0.05); additionally, there were significant interactions between species or isolate and culture medium (Table S2). It should be noted that conidial germination under different culture conditions did not differ significantly (*p* = 0.31). 

**Figure 6:**
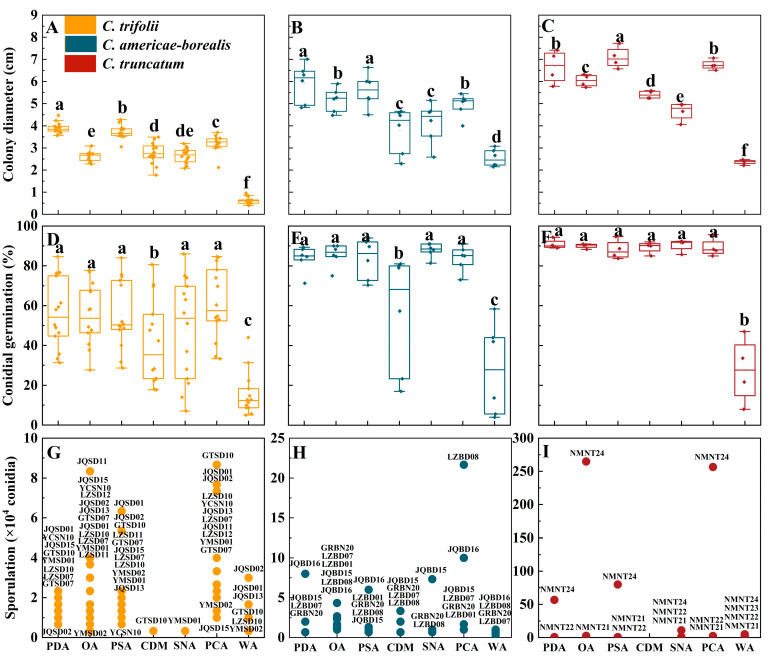
Colony diameter, conidial germination and sporulation for *Colletotrichum* species isolated from alfalfa on different culture media. (**A**–**C**), Colony diameter, (**D**–**F**), Conidial germination for the three *Colletotrichum* species (as identified by color in the legend) on different culture media. Dots for each *Colletotrichum* species are individual isolates. Means with the same letter for a given culture medium (a–f) do not differ significantly among the three *Colletotrichum* species by Tukey’s LSD test (*p* ≥ 0.05). (**G**–**I**), Sporulation. The labels are codes of the isolates, and the data are shown in Table S6. The *X*-axis labels indicate the different culture media: PDA (potato dextrose agar), OA (oatmeal agar), PSA (potato sucrose), CDM (Czapek–Dox medium), SNA (synthetic nutrient-poor agar), PCA (potato sucrose agar) and WA (water agar).

The differences between *Colletotrichum* species for colony diameter with ranking *C. truncatum* > *C. americae-borealis* > *C. trifolii* were confirmed in the testing of growth on different culture media (Figure 6A–C). Among tested media, averaged over isolates, the greatest observed colony diameter of *C. trifolii* was 3.9 cm on PDA, the greatest observed colony diameter of *C. americae-borealis* was 5.9 cm on PDA, and the greatest observed colony diameter of *C. truncatum* was 6.8 cm on PSA. For all three *Colletotrichum* species, the differences in colony diameter between media ranked similarly for the different media, with a general pattern PDA, PSA and PCA > OA, CDM and SNA > WA (Figure 6A–C). 

Except for WA and CDM, the influence of the different media on conidial germination of *C. trifolii* and *C. americae-borealis* was not significant, with the conidial germination ranked as follows: PDA, OA, PSA, SNA, PCA > CDM > WA (Figure 6D,E). The influence of the other media on conidial germination of *C. truncatum* was not significant, with the exception of WA (Figure 6F). The average conidial germination of *C. trifolii* ranged from 15.5% (on WA) to 61.2% (on PCA), while *C. americae-borealis* ranged from 27.9% (on WA) to 87.9% (on SNA), and *C. truncatum* ranged from 27.6% (on WA) to 90.8% (on PDA).

The three tested *Colletotrichum* species produced conidia on all media during the experimental observation period in this research, except for *C. truncatum*, which failed to produce any conidia during the observation of CDM (Figure 6G–I). Across three species, PCA was the best medium for sporulation of *C. americae-borealis* followed by OA, and OA was also good for *C. trifolii* and *C. truncatum*. The highest sporulation of *C. truncatum* was observed on OA, reaching 2.65 × 10^6^ conidia. For *C. americae-borealis,* the highest sporulation was on PCA with 2.2 × 10^5^ conidia, and for *C. trifolii,* the highest sporulation was also on PCA with 8.7 × 10^4^ conidia. Furthermore, there was significant variation in sporulation among different isolates of the same *Colletotrichum* species cultured in identical conditions, for instance, one isolate of *C. truncatum* exhibited maximum sporulation (2.9 × 10^6^ conidia) on OA, while another isolate produced only 2.7 × 10^4^ conidia, and two other isolates failed to produce any spores at all during the trial (Figure 6I). This distribution of sporulation patterns resembled that seen for sporulation of the 24 isolates at varying temperatures.

### 2.6. Pathogenicity

**Figure 7:**
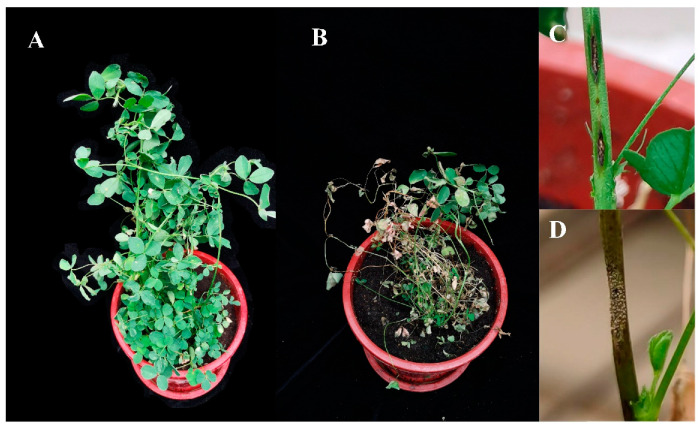
Morphological changes and disease characteristics of alfalfa plants inoculated with *Colletotrichum* species. (**A**), control, no inoculation with *Colletotrichum* species; (**B**), 4 weeks after inoculation with species isolate GTSD10; (**C**), diamond lesion on stem after inoculation with species isolate GTSD10; (**D**), pink conidial clusters on the acervuli after inoculation with species isolate NMNT24. There were no obvious differences between the three *Colletotrichum* species in the symptoms appearing following inoculation.

For the purposes of satisfying Koch’s postulates, it was confirmed that all three *Colletotrichum* species tested produced post-inoculation symptoms matching those seen in field plants (Figure 7), and the morphological characteristics of isolates from the pathogenicity trial were consistent with the inoculated isolates. Also, the three *Colletotrichum* species showed different levels of virulence towards the seven alfalfa varieties. Diseased plants had reduced branch numbers (decreased by 1–4) and branch length (decreased by about 10%), and some branches had become desiccated and withered, in contrast to the control treatment (Figure 7A,B). The stems of inoculated plants displayed typical anthracnose spots, while pink conidial clusters were visible on the acervulus under greenhouse conditions (Figure 7C,D). 

**Figure 8:**
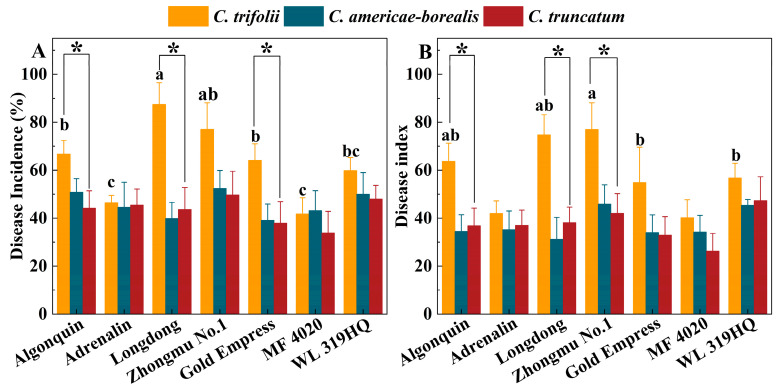
Pathogenicity of three *Colletotrichum* species (isolate GTSD10 for *C. trifolii*, JQBD16 for *C. americae-borealis* and NMNT24 for *C. truncatum*) associated with anthracnose on a range of *Medicago sativa* cultivars 4 weeks after inoculation in a greenhouse, as measured by variation in disease incidence and disease index. (**A**), disease incidence; (**B**), disease index. Means having the same letter do not differ significantly by Tukey’s LSD test (*p* ≥ 0.05). For *C. trifolii* alfalfa variety, means differ significantly at *p* = 0.05 as indicated by letters a, b and c. For *C. americae-borealis* and *C. truncatum*, significance letters are omitted as no difference were detected. Significantly higher susceptibility to *C. trifolii* then to *C. americae-borealis* and *C. truncatum* is indicated by “*”.

The average disease incidence and disease index of the seven alfalfa cultivars inoculated with *C. trifolii* were 63.2% and 58.5, respectively, and these values were significantly higher than those of plants inoculated with *C. americae-borealis* (45.7%, 37.2) and *C. truncatum* (43.2%, 37.2) after four weeks (*p* < 0.05). There was no significant difference between *C. americae-borealis* and *C. truncatum* in terms of incidence and disease index (*p* > 0.05). The disease incidence in Algonquin, Longdong and Gold Empress alfalfa varieties inoculated with *C. trifolii* was significantly (*p* < 0.05) higher than when inoculated with *C. americae-borealis* and *C. truncatum* (Figure 8A). The highest disease incidence (87.4%) was observed in Longdong inoculated with *C. trifolii*, while the disease incidence in the seven cultivars inoculated with *C. americae-borealis* and *C. truncatum* ranged from 33.9% to 52.4%, with no significant difference in incidence between these two *Colletotrichum* species. The disease index of the seven cultivars inoculated with the three *Colletotrichum* species varied from 26.3 to 77.0 (Figure 8B). The disease indexes of Algonquin, Longdong and Zhongmu No. 1 inoculated with *C. trifolii* were significantly (*p* < 0.05) higher than those inoculated with *C. americae-borealis* and *C. truncatum* (Figure 8B). Among these results, the highest disease index of 77.0 was in Zhongmu No. 1, after inoculation with *C. trifolii*, while the highest disease index after inoculation with *C. americae-borealis* and *C. truncatum* was in alfalfa variety Zhongmu No. 1 and WL 319 HQ, where the disease index was 45.9 and 47.3, respectively. Based on the disease incidence and disease index data, the pathogenicity of *C. trifolii* was significantly stronger than that of *C. americae-borealis* and *C. truncatum*.

## 3. Discussion

**Figure 9:**
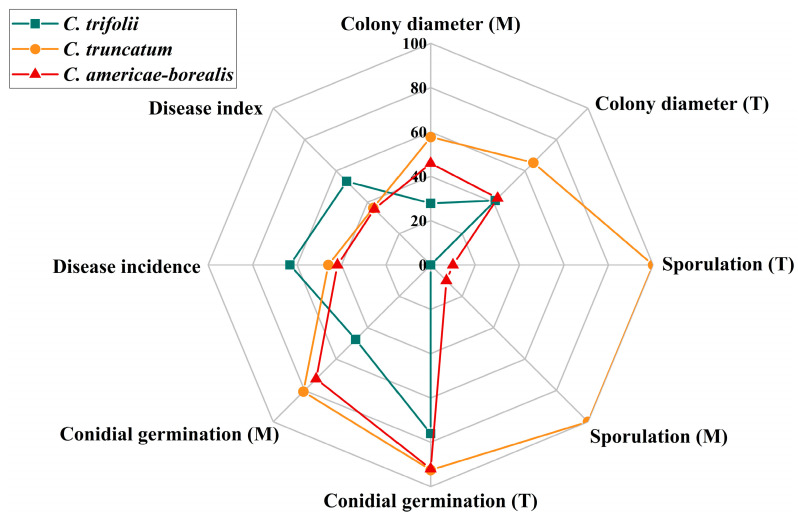
Comparison of three *Colletotrichum* species associated with anthracnose on *Medicago sativa*. M, different culture media; T, different temperatures. Colony diameter is the average colony diameter under different culture media and temperatures, the value is the actual measured value ×10; Sporulation is represented by multiples. One representative isolate (as described in Materials and Methods) was used in the analysis of disease incidence and disease index: GTSD10 for *C. trifolii*, JQBD16 for *C. americae-borealis* and NMNT24 for *C. truncatum*. All isolates were used to assess *Colletotrichum* species differences in colony diameter, conidial germination and sporulation under different temperatures and culture media: 14 isolates for *C. trifolii*, 6 isolates for *C. americae-borealis* and 4 isolates for *C. truncatum*.

Alfalfa anthracnose is one of the most prevalent diseases in major alfalfa-producing areas worldwide. This study presents the first comprehensive survey, to our knowledge, of the *Colletotrichum* species causing alfalfa anthracnose in the main production region of northern China, integrating prevalence, morphological characteristics, phylogenetic analysis and pathogenicity with a comparatively large number of isolates. Through the utilization of multigene phylogenetic and morphological analyses, three distinct *Colletotrichum* species (*C. trifolii*, *C. truncatum* and *C. americae-borealis*) were identified as associated with alfalfa anthracnose. The three species displayed remarkable environmental adaptability, exhibiting a capacity to thrive in temperatures ranging from 4 to 35 °C, with minimal nutrient requirements and pathogenicity towards a wide range of alfalfa varieties. The growth vigor (in terms of colony diameter, sporulation and conidial germination) of *C. truncatum* was the greatest, while the virulence of *C. trifolii* was the strongest (Figure 9). 

*C. trifolii* was identified as the primary pathogen responsible for alfalfa anthracnose, exhibiting not only ubiquitous distribution across plant organs and geographic regions but also a high level of virulence towards alfalfa, as recorded by others [7,27,28]. *C. trifolii* has also been implicated in anthracnose disease among other plant species such as the red clover (*Trifolium pratense* L.), common mallow (*Malva sylvestris* L.) and cluster mallow (*Malva crispa* L.) [29,30,31]. *C. truncatum* was also demonstrated here to be significant as a pathogen responsible for inducing anthracnose on alfalfa, with symptoms characterized by the emergence of large, sunken, irregularly shaped black lesions on mature plant stems, as described by Eken and Demirci [32]. *C. truncatum* has been widely recognized as the primary causative agent of anthracnose in economically important crops such as the soybean (*Glycine max* L.), chili (*Capsicum frutescens* L.), Chinese flowering cabbage (*Brassica parachinensis* L.) and tomato (*Solanum lycopersicum* L.) [33,34,35,36]. *C. americae-borealis* has previously been recognized as an important pathogen of alfalfa anthracnose, once occurring widely in the Xinjiang province of China, with an incidence rate ranging from 7.5% to 53%, while the mortality rate ranged from 0 to 3% [37]. *C. americae-borealis* is known to be also associated with anthracnose among other plant species such as licorice (*Glycyrrhiza uralensis* Fisch.) and oats (*Avena sativa* L.) [38,39]. It is an indisputable fact that many *Colletotrichum* species are associated with alfalfa anthracnose, but alfalfa is not only host to *C. trifolii*, *C. truncatum* and *C. americae-borealis*, which elevates the risk of alfalfa anthracnose when there are susceptible alfalfa cultivars and other hosts in one place. Our findings of distribution and identification of three *Colletotrichum* species present an opportunity for further investigation into their pathogenicity and host range. For example, our data can facilitate the selection of resistant varieties suitable for planting in the field and the design of crop rotations avoiding other susceptible hosts. 

Temperature plays a pivotal role in determining the susceptibility of hosts to microbial invasion and evasion [40]. Temperature influences various stages of *Colletotrichum* species infection, including host surface recognition, spore germination, appressorium formation, hyphal penetration and expansion, and sporulation [41,42]. In this study, the three *Colletotrichum* species were able to accommodate a temperature range of 4 to 35 °C. The ideal temperature for mycelium growth was about 25 °C for *C. trifolii* and *C. americae-borealis*, and 30 °C for *C. truncatum*, while the ideal temperature for conidial germination was about 20 °C for *C. trifolii*, and 25 °C for *C. americae-boreali* and *C. truncatum*. Alfalfa is predominantly cultivated in temperate regions with an optimal growth temperature of 20 to 25 °C [4]; our findings indicate a high degree of coincidence between optimal pathogen growth and conidial germination temperatures and the optimal temperature for host plant growth. Alfalfa plants inevitably faced exposure to infection whenever *Colletotrichum* inoculum was present in the area during the growing season. The above findings also raise the possibility of enhanced prevention and control of alfalfa anthracnose by changing sowing or cutting dates for alfalfa crops in order to avoid the optimal conidial germination and mycelium growth temperatures of *Colletotrichum* species. However, for this approach to be successful, it would be necessary to conduct further studies under field conditions, to ascertain if response thresholds are similar or differ between the laboratory and the field. It would also be necessary to categorize characteristics of locally abundant *Colletotrichum* species and isolates and ascertain whether or not there is management flexibility to alter crop sowing or harvesting dates in ways that could exploit *Colletotrichum* growth responses to improve crop husbandry outcomes.

Moreover, there were intraspecific differences in response to temperature, with not all isolates of same species producing spores at a particular temperature. Hence, despite the similarity in DNA profiles, there exists phenotypic variation among *C. trifolii*, *C. truncatum* and *C. americae-borealis* isolates from alfalfa, even within a localized geographic region. This intraspecific and interspecific variability reported here in the alfalfa infection patterns of *Colletotrichum* and the capacity for infection across a wide of temperature represents a significant disease risk to alfalfa from this pathogenic agent. Indeed, based on our findings, *Colletotrichum* infections can be expected to occur throughout the growing season.

Nutrients are indispensable substrates for biosynthesis and energy release [43]. In the current study, we used a variety of media with different nutrient compositions; the results showed that PDA and PSA were more conducive to mycelial growth, while PCA and OA were more likely to induce sporulation of the three tested *Colletotrichum* species. Compared with PDA and PSA, PCA and OA had a reduced carbon source. We hypothesize that conditions with more nutrients favor mycelium growth, while slightly barren culture conditions favor sporulation. We cannot be sure exactly what factors trigger conidia production, but we suspect that different *Colletotrichum* species have their own adaptations to specific nutrient combinations. In the research of Kim et al. [44], the growth rate of five *Colletotrichum* species on different media differed, and this result was also explained by the different nutritional requirements for different *Colletotrichum* species. Compared with other media, WA prepared with distilled water and agar provided poor nutritional conditions, but it did not adversely affect the completion of the life cycle (conidial germination, mycelium growth and sporulation) of any of the three tested *Colletotrichum* species. This indicates that the tested *Colletotrichum* species are highly adaptable to variation in their growth environment.

The pathogenicity assays conducted in this study showed that the representative isolates of *C. trifolii*, *C. truncatum* and *C. americae-borealis* presented comparable levels of aggressiveness towards a range of alfalfa cultivars. Alfalfa became infected after being sprayed with conidia suspensions, indicating that the *Colletotrichum* species tested in this research can invade an intact host surface directly. This corroborates previous findings that *C. trifolii*, *C. truncatum* and *C. americae-borealis* possess the ability to invade host tissue directly [45,46], although repeating the experiment with a wider range of isolates of each species would be helpful. Another significant outcome of this study was the finding that the seven varieties of alfalfa displayed varying degrees of resistance to three distinct species of *Colletotrichum*, with each variety demonstrating unique susceptibility patterns (Figure 8). Therefore, one approach to managing disease risk in alfalfa field plantings could be through accurate knowledge of the pathogen species and isolates prevalent in particular regions and of varietal susceptibilities, in order to select appropriate alfalfa varieties. 

## 4. Materials and Methods

### 4.1. Survey and Sampling of Colletotrichum Species

An extensive investigation of anthracnose infections in alfalfa crops was carried out throughout the major alfalfa-producing regions of northern China in July and October 2020. The surveyed regions encompassed nine counties across five provinces: Yongchang, Linze, Gaotai, Suzhou, Yumen (in Gansu province), Helan (Ningxia), Fugu (Shaanxi), Huanghua (Hebei) and Arhorchin (Inner Mongolia). Fields of monoculture alfalfa more than 660 m^2^ in area were chosen as survey sites (Figure 1), and information on alfalfa cultivation and management at the selected sites was collated (Table 1). In each field where disease was found, four 1 m^2^ plots were randomly selected to quantify the occurrence of anthracnose. The number of healthy and visibly infected plants in each sample were recorded, and the proportion of the diseased plants to total plants was recorded as a measure of disease incidence. Disease severity was assessed using the scale developed by Welty [47], where 0 = healthy with no lesions; 1 = small, nearly circular spots of necrotic black tissue; 2 = spots elongating vertically and becoming lesions, but non-sporulating; 3 = diamond-shaped or oval-shaped lesions with acervuli but not girdling the stem; 4 = stem-girdling lesions with leaves wilting; 5 = dead plant. Disease index was calculated as

(1) $$\mathrm{Disease}\;\mathrm{index}=\frac{\sum (S_{i}\times N_{i})}{N\times 5}\times 100$$

where *S_i_* is the disease severity, *N_i_* is the number of diseased plants with a given level of disease severity (*i* = 0 ... 5), and *N* is the total number of surveyed plants, and the maximum possible value of the disease index is 100.

Five to ten stems and leaves exhibiting anthracnose symptoms were collected from each surveyed field and brought back to the laboratory via a specimen holder for the purpose of isolating and identifying *Colletotrichum* species.

### 4.2. Isolation and Maintenance of the Pathogen

The collected samples underwent a thorough cleansing with tap water to eliminate any surface soil and other substances, before being randomly divided into several 5 mm sections at the junction of the diseased and healthy tissue using disinfected scalpels. These fragments underwent surface sterilization in a solution of 1% sodium hypochlorite for 60 s, followed by immersion in 75% alcohol for 30 s; finally, they were subjected to a thorough rinsing process with sterile distilled water, undergoing three to four cycles before being carefully dried on using sterilized filter paper. Fragments were inoculated onto fresh PDA and incubated at 25 °C in the dark. Observations were made daily to monitor the emergence of fungal colonies, and colonies resembling *Colletotrichum* in morphology were transferred to new PDA. The colony color and acervuli emergence were observed with the naked eye, and conidia size, shape and color, and setae emergence were observed using an optical microscope (Olympus BX51, Tokyo, Japan). Single-spore techniques, described as Cai et al. [48], were applied to obtain pure isolates. All the isolates were preserved on a slant of PDA at 4 °C for further investigation. 

### 4.3. DNA Extraction, Amplification and Sequencing

The isolates resembling *Colletotrichum* species were cultured on fresh PDA for 5 days in the dark at 25 °C, specifically for the purpose of genomic DNA extraction. The fresh mycelium was carefully scraped off the surface of the colony using a sterilized glass slide and subsequently subjected to DNA extraction utilizing the Ezup Column Fungi Genomic DNA Purification Kit (Sangon Biotech, Shanghai, China), following the manufacturer’s protocol. The DNA concentration was measured using the Nanodrop 2000 spectrophotometer (Thermo Scientific, Waltham, MA, USA) and manually adjusted to 100 ng·μL^−1^. The *ITS*, *GAPDH*, *ACT* and *HIS3* genes were amplified using the primers ITS–1F and ITS–4R for *ITS* [23,44,49], GDF1 and GDR1 for *GAPDH* [44,49], ACT–512F and ACT–783R for *ACT* [23,49], and CYLH–3F and CYLH–3R for *HIS3* [23], respectively. The primer nucleotide sequences used in this study are listed in Table S3. 

The PCR reactions were conducted in a final volume of 25 μL, comprising 12.5 μL Taq DNA polymerase mix (Sangon Biotech), 1.0 μL DNA template, 9.5 μL ddH_2_O and 1.0 μL of forward and reverse primer (10 μmol·L^−1^) on the 2720 Thermal Cycler (Biosystems, Waltham, MA, USA). The thermocycler was programmed with the following parameters: an initial denaturation step of 4 min at 94 °C, followed by 35 cycles of 30 s at 95 °C, annealing for 30 s, extension of 45 s at 72 °C, and a final extension step at 72 °C for 7 min. The annealing temperatures used for different primers were 54 °C for *ITS*, 52 °C for *HIS3*, and 56 °C for *ACT* and *GAPDH*. The purification and paired-end of Illumina sequencing of PCR products were performed by Sangon Biotech Company (Shanghai, China).

### 4.4. Sequence Alignment and Phylogenetic Analyses

The sequence data were automatically assembled using the “Sequence assembly” program of DNAMAN X (USA). The assembled sequences were used as query sequences for searching against the NCBI (National Center for Biotechnology Information, Bethesda, MD, USA) nucleotide database, and then, we submitted and obtained accession numbers from GenBank. Reference sequences were obtained from NCBI GenBank (Table S4). The nucleotide sequence of the 24 isolates from alfalfa and reference sequences of other *Colletotrichum* species were aligned using the “ClustalW” method implemented in MEGA X, and the alignments were manually edited [50]. The processed sequences were spliced in the following order: “*ITS*–*GAPDH*–*HIS3*–*ACT*”. Evolution models were estimated in “MODELS” of MEGA X, and the robustness of the resulting trees was assessed through 1000 bootstrap replications using the “TN93 + G” model. The multi-locus alignment was employed to construct maximum parsimony and maximum likelihood phylogenetic trees, respectively. The tree was visualized using Microsoft word 365 and FigTree v1.4.4.

### 4.5. Morphological Observation and Biological Characterization

The morphological characteristics of isolates on PDA, including colony size and color, conidia size, and setae color and length, were recorded over a 7-day incubation period at 25 °C in the dark [51]. The sizes of 50 conidia, 50 setae and 50 appressoria were measured for each isolate through random selection using an optical microscope (Olympus BX51, Tokyo, Japan). Appressoria were induced and produced using a slide method described by Xue et al. [52]. 

For the 24 *Colletotrichum* isolates obtained in this study, mycelium growth, sporulation and conidial germination were compared at eight different temperatures. The series of temperature comparisons was carried out using PDA (9 cm plastic Petri dishes containing 15 mL of PDA, produced by LABGIC Co., Ltd., Beijing, China), where 1 mm diameter mycelium fragments were inoculated onto fresh culture media and incubated in darkness at temperatures of 4, 10, 15, 20, 25, 30, 35 and 40 °C. We used four incubators. Hence, four of the eight test temperatures were randomly allocated to the four incubators and a batch of culture medium prepared sufficient for 384 plates (4 incubators, each containing the 24 *Colletotrichum* isolates and 4 biological replicates of each). The procedure was then repeated with a second batch of culture medium for a further 384 plates to test the remaining four temperatures from the temperature series in the same four incubators, as shown in Table S5. A thermometer was placed in each incubator to ensure that the temperature of the incubator was consistent with the designated test temperature. In the experiment design adopted, each incubator was always allocated all four replicates of all 24 *Colletotrichum* isolates tested, and great professional care was taken to keep culture conditions identical (apart from incubator temperature settings) in both batches and all four incubators.

After seven days of incubation, the colony diameter of the 24 isolates from the three *Colletotrichum* species was measured in two intersecting directions. After measuring colony diameter, sporulation on the same plates was assessed by mechanically disturbing the colony using a sterilized glass slide with 5 mL sterile distilled water to suspend spores. The mixture of mycelium and spores was filtered through sterile gauze, and the conidia count was ascertained by a hemocytometer observed under a light microscope [53]. The spore suspension from each plate was counted three times and the counts averaged. The total number of spores extracted from each culture plate was estimated as *n* × 10^4^ × *V*, where *n* = the average of five counts of spores in a 0.1 mm^3^ hemocytometer counting chamber, 10^4^ is a constant representing the number of 0.1 mm^3^ counting volumes per mL, and *V* is the water volume used to wash spores from the culture plate, in this case, 5 mL.

In a new series of experiments, conidial germination was assessed at the same eight temperatures ranging from 4 °C to 40 °C as described above, by quantifying the number of germinated conidia from conidia suspensions. The agar slide method was employed to evaluate conidial germination [54]. A sterile slide was immersed in sterilized PDA at approximately 60 °C and slowly taken out from the medium and left to cool. After agar solidification, the culture medium on the opposite side of the sterile slide was discarded, and the front of the side was utilized for conidial germination testing. These slides were placed in a sterile Petri dish, and sterile distilled water was added to maintain humidity inside the Petri dish. Using a pipette, 10 μL aliquots of conidia suspension (10^6^ spores·mL^−1^) were dispensed onto an agar slide and spread using a wire loop. Conidial germination was investigated by inoculating PDA agar slides and incubating them in the dark, the experimental design for the series of temperature-test experiments again followed the plan in Table S5. After 24 h, the number of germinated spores among a sample of 100 randomly selected spores was counted using a light microscope. A spore was considered to have germinated if its primary germ tube length was more than half of its width [53]. There were three replicates of each isolate.

The effect of culture media on mycelium growth, sporulation and conidial germination of the 24 isolates was also assessed. The series of culture media comparisons was carried out using potato sucrose agar (PSA) [55], oatmeal agar (OA) [21], synthetic nutrient poor-agar (SNA, produced by Shandong TOPBIO Co., Ltd., Shandong, China) [21], Czapek–Dox medium (CDM) [56], potato carrot agar (PCA) [15], water agar (WA) [55] and PDA, where 1 mm diameter mycelium fragments were inoculated onto fresh culture media and incubated in darkness at temperatures of 25 °C. The Petri dishes related to the above test were 9 cm in diameter, each containing 15 mL of medium. After seven days of incubation, the colony diameter of the 24 isolates was measured in two intersecting directions. The sporulation on each plate was measured and calculated as previously mentioned. The effect of different media on the germination of conidia from each isolate was measured by inoculating slides coated with the different media, with the respective 10 μL aliquots of conidia suspension (10^6^ spores·mL^−1^) of each isolate, and placing them in a constant-temperature incubator at 25 °C in the dark. The sterile slides of different culture media were prepared according to the method at different temperatures. After 24 h, the number of germinated spores among a sample of 100 randomly selected spores was counted using a light microscope. There were three replicates of each isolate.

### 4.6. Pathogenicity 

The pathogenicity of *Colletotrichum* species isolated from alfalfa was evaluated on seven cultivars of *Medicago sativa* (cv. Algonquin, Adrenalin, Longdong, Zhongmu No. 1, Gold Empress, MF 4020, and WL 319HQ) in a greenhouse. Representative isolates of each species (NMNT24 for *C. truncatum*, JQBD16 for *C. americae-borealis*, GTSD10 for *C. trifolii*) were used to inoculate 4-week-old seedlings (6–10 leaves). The representative isolates were chosen based on multiple criteria, including prolific sporulation, the temperature range and culture media for which sporulation was observed, and the criterion that there was conidia production from acervuli. The alfalfa seeds were subjected to surface sterilization in 75% alcohol for a duration of 3 min; then they were washed three times in sterilized water and transferred into sterilized Petri dishes with a double-layer filter paper to facilitate germination. After 48 h, imbibed seeds of uniform size were selected and transplanted into 10 cm diameter pots filled with sterilized substrate (produced by Pindstrup, Denmark) and sand mixed in a 3:1 ratio by volume. The plants were cultivated in a greenhouse for four weeks. The experiment was laid out as a completely randomized design with three replicates of four treatments (three *Colletotrichum* species and the control). Each 10 cm diameter pot-replicate contained five plants. 

The inoculum used was a conidia suspension (10^6^ spores·mL^−1^) from three *Colletotrichum* species that had been grown on PDA for one month. The inoculation was performed by spraying the stem and leaf, so as to create a mist of water droplets on the leaf surface without allowing coalescence into larger droplets. Black plastic bags were used to cover the plants to maintain high humidity conducive to pathogen infection and removed after 48 h. Then, the plants remained in the greenhouse for four weeks. The control plants were sprayed with distilled water, while the other operations were as previously described. Pathogenicity was evaluated based on disease incidence (the proportion of the total number of stems per pot that were diseased) and disease index, recorded as previously described [17]. In order to test Koch’s postulates, a diseased plant was randomly selected in each pot for isolation and morphological observation. The experiment was repeated twice.

### 4.7. Statistical Analysis

The disease incidence and index were expressed as the average ± SD. On conducting the Shapiro–Wilk’s tests in Q-Q Plots (α = 0.05) using the SPSS 22 Statistics package (SPSS Inc., Chicago, IL, USA), we found that our data were normally distributed, and we therefore proceeded to perform ANOVA without data transformation. The colony diameter, conidial germination and sporulation of each isolate from the same *Colletotrichum* species at different temperatures or on different culture media, and disease incidence and index from pathogenicity testing were subjected to one-way ANOVA using the SPSS 22 Statistics package. Tukey’s multiple range test was used to test post hoc multiple comparisons (equal variances assumed), and the Tamhane’s T2 test was used if the variances across temperature or media were not homogeneous (*p* < 0.05). The factors species, isolate and culture medium, and their interactions for colony diameter, conidial germination and sporulation were analyzed using the Nested ANOVA command in the SPSS 22 Statistics package and Type II sums of squares. The factor temperature was not included in the ANOVA and was not statistically tested due to the confounding among temperature, incubator, and culture batch effects in the experimental design. Graphs were generated using Origin 2023 (OriginLab Crop., Northampton, MA, USA).

## 5. Conclusions

In summary, alfalfa anthracnose was a prevalent phenomenon in northern China and the main pathogens responsible for it were identified as *C. trifolii*, *C. truncatum* and *C. americae-borealis* based on their distinctive morphological and molecular characteristics. These isolates exhibited excellent adaptation to a wide range of temperature (from 4 to 35 °C) and low nutritional conditions, as evidenced by their optimum mycelial growth, conidial germination and sporulation. Furthermore, pathogenicity assays revealed that three species demonstrated virulence towards multiple alfalfa varieties, with *C. trifolii* having the most aggressive behavior. The findings showed that the *Colletotrichum* species investigated in this study possessed a remarkable adaptability to the environment, and the representative isolates of three species exhibited high virulence towards alfalfa varieties, thereby posing a significant challenge for disease control and resistance breeding against alfalfa anthracnose. Our findings have led to new insights into the pathogen and distribution of alfalfa anthracnose and contribute to the development of appropriate management strategies for controlling alfalfa anthracnose, thereby enhancing the agricultural productivity and sustainability of high-quality forage. A follow-up study to test for differences in pathogenicity between *Colletotrichum* isolates would be helpful.

## Figures and Tables

**Table 1:** Alfalfa cultivation and management information for sampled stands.

Location	Main Varieties	Years Established	Phenological Stage(s)	Neighboring Crops
Helan	Adrenalin, MF 4020	1	Late bud, Early flower	corn, soybean, others
Fugu	Gibraltar	4, 3, 2	Late flower	corn, others
Huanghua	Zhongmu No. 1	5	Early flower	wheat, others
Arhorchin	4015, Adrenalin, Xinmu No. 3	3, 1	Late bud, Early flower	oat, corn,
Yongchang	unknown	1	Late bud	corn, wheat
Linze	WL 363HQ; WL 319HQ	2	Late flower	corn, wheat
Gaotai	Zhongmu No. 1	1	Early flower	corn, soybean, others
Suzhou	Zhongmu No. 1, Gannong No. 3	5, 1	Early flower	corn, soybean, wheat, others
Yumen	Gannong No. 3, Algonquin, Gold Empress, Adrenalin	6, 5	Early flower	corn, soybean, wheat, others

Others: Crops of Leguminosae, Poaceae, Brassicaceae and Solanaceae.

**Table 2:** Disease incidence of alfalfa anthracnose in Northern China.

Location of Field	Province	Anthracnose Occurrence	Mean Disease Incidence (%)	Mean Disease Index
Helan	Ningxia	Samplings ^a^	- ^b^	-
Fugu	Shaanxi	N ^c^	-	-
Huanghua	Hebei	N	-	-
Arhorchin	Inner Mongolia	Samplings	-	-
Yongchang	Gansu	Y ^d^	9 ± 2.6 ^e^	5 ± 1.3
Linze	Gansu	Y	20 ± 12.1	10 ± 5.2
Gaotai	Gansu	Y	20 ± 4.8	12 ± 2.5
Suzhou	Gansu	Y	45 ± 10.8	17 ± 4.5
Yumen	Gansu	Y	13 ± 1.9	9 ± 0.9

^a^ Anthracnose occurred sporadically and some *Colletotrichum* isolates were collected; ^b^ Not calculated; ^c^ N, no anthracnose symptoms; ^d^ Y, anthracnose occurred in the fields; ^e^ The values represent the average ± standard error.

## Data Availability

Data are contained within the article and Supplementary Materials.

## Supplementary Materials

The following supporting information can be downloaded at: https://www.mdpi.com/article/10.3390/plants13131780/s1. Table S1: Monthly weather conditions during the investigation; Table S2: Variance analysis for factors and their interaction for colony diameter, sporulation and conidial germination on different culture media; Table S3: List of primers used for PCR amplification and sequencing in this study; Table S4: GenBank accession numbers of *Colletotrichum* isolates in this study (in bold) and the representative *Colletotrichum* species for phylogenetic analysis; Table S5: The experimental design used when evaluating mycelium growth, sporulation and conidial germination of 24 isolates at different temperature; Table S6: The data for colony diameter, sporulation and conidial germination at different temperatures and different culture media.
