# Supplementary material for: A Biological Comparison of Three Colletotrichum Species Associated with Alfalfa Anthracnose in Northern China
Source: Plants (Basel). 2024 Jun 27;13(13):1780. doi: 10.3390/plants13131780 (PMC11244077; doi:10.3390/plants13131780)
Supplement: Supplementary file 1 [file plants-13-01780-s001.zip › Table S1-S5.pdf]

Supplementary materials

Table S1. Monthly weather conditions during the investigation

Location	July			October		
	T_{max} (℃)	T_{man} (℃)	Rainfall	T_{max} (℃)	T_{min} (℃)	Rainfall
Helan	32	19	26.6	-	-	-
Fugu	29	18	150.5	-	-	-
Huanghua	32	23	71.7	-	-	-
Arhorchin	31	19	44.4	-	-	-
Yongchang	26	13	44.8	15	5	58.0
Linze	31	18	14.3	19	7	4.7
Gaotai	32	17	35.4	19	6	3.0
Suzhou	29	19	1.6	18	7	1.7
Yuman	30	17	3.9	16	5	0

T_{max}, the average maximum daily temperature; T_{min}, the average minima daily temperature.

Table S2. Variance analysis for factors and their interaction for colony diameter, sporulation and conidial germination on different culture media

Factors	*df*	Colony diameter		Sporulation		Conidial germination	
		F	*P*	*F*	*P*	*F*	*P*
S	2	287.34	<0.001	18.48	<0.001	18.48	<0.001
I	21	162.31	<0.001	19.41	<0.001	122.21	<0.001
C	6	4.65	<0.001	2.34	0.03	2.342	0.31
I×C	126	13.03	<0.001	4.17	<0.001	36.42	<0.001
S×C	12	22.57	<0.001	4.77	<0.001	4.77	<0.001

Note: S, *Colletotrichum* species, *C. truncatum*, *C. trifolii*, and *C. americae-borealis*, respectively; I, isolates, a total of 24 isolates; C, medium, PDA, PSA, OA, CDM, SNA, PCA, and WA, respectively.

Table S3. List of primers used for PCR amplification and sequencing in this study

Gene	Primer	Sequence (5'-3')	Annealing temperature (℃)	Reference
ITS	ITS-1	TCC GTA GGT GAA CCT GCG G	54℃	[23, 48, 49]
	ITS-4	TCC TCC GCT TAT TGA TAT GC		
HIS3	CYLH-3F	AGG TCC ACT GGT GGC AAG	52℃	[23]
	CYLH-3R	AGC TGG ATG TCC TTG GAC TG		
ACT	ACT-512F	ATG TGC AAG GCC GGT TTC GC	56℃	[23, 48]
	ACT-783R	TAC GAG TCC TTC TGG CCC AT		
GAPDH	GDF	GCC GTC AAC GAC CCC TTC ATT GA	56℃	[48, 49]
	GDR	GGG TGG AGT CGT ACT TGA GCA TGT		

Table S4. GenBank accession numbers of *Colletotrichum* isolates in this study (in bold) and the representative *Colletotrichum* species for phylogenetic analysis

Species	Isolate	Host	Locality	GenBank accession number			
				ITS	*HIS3*	*ACT*	*GAPDH*
C. aciculare	MFLU 13-0280	unknown	Thailand	KT290265	……	KT290250	KT290254
C. acidae	MFLU 18-0233	*Phyllanthus acidus*	Thailand	MG996506	……	MH003698	MH003692
C. antirrhinicola	CBS 102189*	*Antirrhinum majus*	New Zealand	KM105180	KM105320	KM105390	KM105531
C. americae-borealis	CBS 136232*	*Medicago sativa*	USA	KM105224	KM105364	KM105434	KM105579
C. americae-borealis	CBS 136855	*Medicago sativa*	USA	KM105225	KM105365	KM105435	KM105580
C. americae-borealis	**JQBD15**	***Medicago sativa***	**China**	**OQ975307**	**OR762941**	**OR762965**	**OR762989**
C. americae-borealis	**JQBD16**	***Medicago sativa***	**China**	**OQ975308**	**OR762942**	**OR762966**	**OR762990**
C. americae-borealis	**LZBD07**	***Medicago sativa***	**China**	**OQ975309**	**OR762943**	**OR762967**	**OR762991**
C. americae-borealis	**LZBD08**	***Medicago sativa***	**China**	**OQ975310**	**OR762944**	**OR762968**	**OR762992**
C. americae-borealis	**LZBD01**	***Medicago sativa***	**China**	**OQ975311**	**OR762945**	**OR762969**	**OR762993**
C. americae-borealis	**GRBN20**	***Medicago sativa***	**China**	**OQ975312**	**OR762946**	**OR762970**	**OR762994**
C. atractylodicola	KACC 47873	*Ageratina altissima*	South Korea	OR431680	OR449486	OR449417	OR449467
C. bidentis	COAD 1020*	*Bidens subalternans*	Brazil	KF178481	KF178554	KF178578	KF178506
C. bryoniicola	CBS 109849*	*Bryonia dioica*	Netherlands	KM105181	KM105321	KM105391	KM105532
C. curcumae	IMI 288937*	*Curcuma longa*	India	GU227893	GU228089	GU227991	GU228285
C. destructivum	CBS 157.83	*Medicago sativa*	Serbia	KM105215	KM105355	KM105425	KM105570
C. destructivum	CBS 167.58	*Medicago sativa*	Italy	KM105213	KM105353	KM105423	KM105568
C. destructivum	CBS 136228*	*Trifolium hybridum*	USA	KM105207	KM105347	KM105417	KM105561
C. fuscum	CBS 133704	*Digitalis dubia*	Netherlands	KM105176	KM105316	KM105386	KM105526
C. fuscum	CBS 133701*	*Digitalis lutea*	Germany	KM105174	KM105314	KM105384	KM105524
C. fusiforme	MFLU 13-0291*	unknown	Thailand	KT290266	……	KT290251	KT290255
C. galinsogae	KACC 43120	*Galinsoga quadriradiata*	South Korea	OR431670	OR449487	OR449421	OR449477
C. higginsianum	IMI 349061*	*Brassica Trin*	Tobago	KM105184	KM105324	KM105394	KM105535
C. higginsianum	Abr 2-2	*Brassica Trin*	Tobago	KM105188	KM105328	KM105398	KM105540

C. jasiminigenum	LC923	*Jasminum sambac*	Vietnam	HM131513	……	HM131508	HM131499
C. lentis	CBS 127605	*Lens culinaris*	Canada	KM105241	KM105381	KM105451	KM105598
C. lindemuthianum	CBS 144.31*	*Phaseolus vulgaris*	Germany	JQ005779	JQ005821	JQ005842	JX546712
C. lindemuthianum	CBS 143.31	*Phaseolus vulgaris*	Germany	JX546808	JX546760	JX546616	JX546611
C. lini	CBS 136856	*Medicago sativa*	USA	KM105233	KM105373	KM105443	KM105589
C. malvarum	CBS 123.24	*Malvaceae*	unknown	KF178479	KF178552	KF178576	KF178503
C. neorubicola	CCR144	*Rubus idaeus*	China	MK529906	……	MK547523	MK547520
C. ocimi	CBS 298.94*	*Ocimum basilicum*	Italy	KM105222	KM105362	KM105432	KM105577
C. orbiculare	CBS 122.24	*Cucumis satavus*	UK	KF178467	KF178540	KF178564	KF178492
C. orbiculare	CBS 274.54	*Cucumis satavus*	Netherlands	KF178462	KF178535	KF178559	KF178486
C. panacicola	KACC 410614	*Panax ginseng*	South Korea	OR880098	OR885835	OR885814	OR885828
C. pisicola	CBS 724.97	*Pisum sativum*	USA	KM105172	KM105312	KM105382	KM105522
C. pleopeltidis	CPC 39342	*Pleopeltis* sp.	South Africa	MW883412	……	MW890024	……
C. shisoi	KACC 40894	unknown	South Korea	OR431665	OR449488	OR449422	OR449462
C. sidae	CBS 504.97*	*Sida spinosa*	USA	KF178472	KF178545	KF178569	KF178497
C. sidae	CBS 518.97	*Sida spinosa*	USA	KF178471	KF178544	KF178568	KF178496
C. spinosum	CBS 515.97*	*Xanthium spinosum*	Australia	KF178474	KF178547	KF178571	KF178498
C. subacidae	NN071131	*Ailanthus*	China	MZ595910	MZ673930	MZ664208	MZ664072
C. tabaci	CBS 124249	unknown	unknown	KM105206	KM105346	KM105416	KM105560
C. tanaceti	TAS060-0004	*Tanacetum cinerariifolium*	Australia	JX218230	……	JX218240	JX218245
C. tebeestii	CBS 522.97*	*Malva pusilla*	Canada	KF178473	KF178546	KF178570	KF178505
C. trifolii	CBS 158.83*	*Trifolium*	USA	KF178478	KF178551	KF178575	KF178502
C. trifolii	CBS 128554	*Medicago sativa*	USA	KF178476	KF178549	KF178573	KF178500
C. trifolii	**YMSD01**	***Medicago sativa***	**China**	**OQ553751**	**OR762951**	**OR762975**	**OR762999**
C. trifolii	**YMSD02**	***Medicago sativa***	**China**	**OQ971753**	**OR762952**	**OR762976**	**OR763000**
C. trifolii	**JQSD15**	***Medicago sativa***	**China**	**OQ971754**	**OR762953**	**OR762977**	**OR763001**
C. trifolii	**JQSD01**	***Medicago sativa***	**China**	**OQ971755**	**OR762954**	**OR762978**	**OR763002**
C. trifolii	**JQSD02**	***Medicago sativa***	**China**	**OQ971756**	**OR762955**	**OR762979**	**OR763003**
C. trifolii	**JQSD11**	***Medicago sativa***	**China**	**OQ971757**	**OR762956**	**OR762980**	**OR763004**

C. trifolii	**JQSD13**	***Medicago sativa***	**China**	**OQ971758**	**OR762957**	**OR762981**	**OR763005**
C. trifolii	**GTSD07**	***Medicago sativa***	**China**	**OQ971759**	**OR762958**	**OR762982**	**OR763006**
C. trifolii	**GTSD10**	***Medicago sativa***	**China**	**OQ971760**	**OR762959**	**OR762983**	**OR763007**
C. trifolii	**LZSD07**	***Medicago sativa***	**China**	**OQ971761**	**OR762960**	**OR762984**	**OR763008**
C. trifolii	**LZSD10**	***Medicago sativa***	**China**	**OQ971762**	**OR762961**	**OR762985**	**OR763009**
C. trifolii	**LZSD11**	***Medicago sativa***	**China**	**OQ971763**	**OR762962**	**OR762986**	**OR763010**
C. trifolii	**LZSD12**	***Medicago sativa***	**China**	**OQ971764**	**OR762963**	**OR762987**	**OR763011**
C. trifolii	**YCSN10**	***Medicago sativa***	**China**	**OQ971765**	**OR762964**	**OR762988**	**OR763012**
C. truncatum	CBS 151.35	*Phaseolus lunatus*	USA	GU227862	GU228058	GU227960	GU228254
C. truncatum	CBS 260.85	*Crotalaria spectabilis*	USA	GU227875	GU228071	GU227973	GU228267
C. truncatum	CBS 182.52	*Glycine max*	USA	GU227866	GU228062	GU227964	GU228258
C. truncatum	**NMNT21**	***Medicago sativa***	**China**	**OQ975302**	**OR762947**	**OR762971**	**OR762995**
C. truncatum	**NMNT22**	***Medicago sativa***	**China**	**OQ975303**	**OR762948**	**OR762972**	**OR762996**
C. truncatum	**NMNT23**	***Medicago sativa***	**China**	**OQ975304**	**OR762949**	**OR762973**	**OR762997**
C. truncatum	**NMNT24**	***Medicago sativa***	**China**	**OQ975305**	**OR762950**	**OR762974**	**OR762998**
C. utrechtense	CBS 130243*	*Trifolium pratense*	Netherlands	KM105201	KM105341	KM105411	KM105554
C. utrechtense	CBS 135827	*Trifolium pratense*	Netherlands	KM105202	KM105342	KM105412	KM105555
C. vignae	CBS 501.97	*Vigna unguiculata*	Nigeria	KM105183	KM105323	KM105393	KM105534
C. vignae	IMI 334960	*Vigna unguiculata*	Nigeria	KM105182	KM105322	KM105392	KM105533
Monilochaetes infuscans	CBS 869.96	*Ipomoea batatas*	South Africa	JQ005780	JQ005822	JQ005843	JX546612

*ex-holotype, ex-epitype or ex-neotype culture. *ITS*: internal transcribed spacers and intervening 5.8S nrDNA; *HIS3*: partial histone H3 gene; *ACT*: partial actin gene; *GAPDH*: partial glyceraldehyde-3-phosphate dehydrogenase gene. Sequences generated in this study are indicated in bold.

Table S5. The experimental design used when evaluating mycelium growth, sporulation and conidial germination of 24 isolates at different temperature

	Incubator 1	Incubator 2	Incubator 3	Incubator 4
Batch 1	4℃	35℃	40℃	15℃
	24 (isolates) ×4 (replicates)	24 (isolates) ×4 (replicates)	24 (isolates) ×4 (replicates)	24 (isolates) ×4 (replicates)
Batch 2	20℃	10℃	25℃	30℃
	24 (isolates) ×4 (replicates)	24 (isolates) ×4 (replicates)	24 (isolates) ×4 (replicates)	24 (isolates) ×4 (replicates)
